# Accidents domestiques chez les enfants âgés de 0 à 15 ans au Service des urgences du Centre hospitalier national El-Maarouf (Comores) de janvier à février 2021

**DOI:** 10.11604/pamj.2026.53.65.46757

**Published:** 2026-02-06

**Authors:** Azhar Salim Mohamed, Attoumane Fahad, Alou Diaby, Said Hassani Kourachia, Charaf Mohamed, Mohamed Hachim, Rouaidat Izdine Soule, Gabriel Ngom

**Affiliations:** 1 Médecine du Travail, Moroni, Union des Comores,; 2 Centre Hospitalier National El-Maarouf, Moroni, Union des Comores,; 3 Institut de Formation et de Recherche en Population, Développement et Santé de la Reproduction, Dakar, Sénégal,; 4 Direction Régionale de la Santé de Ngazidja, Moroni, Union des Comores,; 5 Service de Chirurgie Pédiatrique, Université Cheikh Anta Diop, Dakar, Sénégal,; 6 Service de Chirurgie Orthopédique et Traumatologie, Dakar, Sénégal

**Keywords:** Accidents domestiques, enfant, chute, urgences, Moroni, Comores, Domestic accidents, children, falls, emergency department, Moroni, Comoros

## Abstract

**Introduction:**

un accident de la vie courante (AcVC) se définit comme un traumatisme non intentionnel qui n'est ni un accident de la circulation ni un accident du travail. Les accidents domestiques (AD) sont ceux qui se produisent à la maison ou dans ses alentours immédiats. Ce travail avait pour but de rapporter les aspects sociodémographiques et étiopathogéniques des AD aux urgences du Centre hospitalier national (CHN) El-Maarouf de Moroni.

**Méthodes:**

nous avons mené une étude rétrospective descriptive s'étendant du 1^er^ janvier au 28 février 2021. Nous avons inclus tous les enfants de moins de 15 ans reçus et pris en charge au service des urgences. Nous avons étudié divers paramètres sociodémographiques tels que l'âge, le genre, l'origine géographique des patients et les caractéristiques de l'accident: leur fréquence, le lieu de survenue, le type et le mécanisme.

**Résultats:**

nous avons colligé au total 223 AD durant notre période d'étude, soit une fréquence de 11,17%. Les garçons étaient les plus touchés avec 65,02% des cas. La moyenne d'âge était de 7,07 ans. La tranche d'âge des 0 à 5 ans était la plus représentée avec 43,50% des cas. Selon le type d'accident, les traumatismes venaient en première position avec 80,27% des cas suivis des ingestions de corps étrangers (CE) et des brûlures. La chute était à l'origine de la majorité des traumatismes avec 88,27%. S'agissant des ingestions de CE, les pièces de monnaie et les objets métalliques représentaient près de la moitié des cas. Les brûlures étaient plus souvent dues à un liquide chaud, suivies des flammes.

**Conclusion:**

les AD chez l'enfant sont fréquents dans notre service et surviennent essentiellement dans la cour et au salon. Les victimes sont essentiellement des garçons âgés de moins de 5 ans. Il s'agissait le plus souvent de traumatismes ou d'ingestion de CE.

## Abstract (en)

**Introduction:**

a home and leisure accident (HLA) is defined as an unintentional injury that is neither a road traffic accident nor an occupational accident. Domestic accidents (DAs) are those that occur at home or in its immediate surroundings. The purpose of this study was to report the sociodemographic and etiopathogenic aspects of domestic accidents seen in the Emergency Department of the CHN El-Maarouf in Moroni.

**Methodology:**

we conducted a retrospective descriptive study from January 1 to February 28, 2021. All children under 15 years of age who were admitted to and treated in the emergency department were included. We analyzed various socio-demographic parameters such as age, sex, and geographic origin of patients, as well as accident characteristics: frequency, place of occurrence, type, and mechanism.

**Results:**

a total of 223 domestic accidents were recorded during the study period, representing a frequency of 11.17%. Boys were the most affected, accounting for 65.02% of cases. The mean age was 7.07 years. The 0 to 5-year age group was the most represented, with 43.50% of cases. According to the type of accident, trauma was the most common, accounting for 80.27% of cases, followed by ingestion of foreign bodies and burns. Falls were responsible for the majority of trauma cases, accounting for 88.27%. Concerning foreign body ingestions, coins and metallic objects represented nearly half of the cases. Burns were most often caused by hot liquids, followed by flames.

**Conclusion:**

domestic accidents in children are common in our department and occur mainly in the yard and the living room. The victims were predominantly boys under the age of 5. The most common causes were traumatic injuries and ingestion of foreign bodies.

## Introduction

Un accident de la vie courante (AcVC) se définit comme un traumatisme non intentionnel qui n'est ni l’accident de la circulation ni l’accident du travail. Les accidents domestiques (AD) sont ceux qui se produisent à la maison ou dans ses alentours immédiats. Chez l'enfant, les AD ont fait l'objet de plusieurs travaux scientifiques dans le monde [1-6]. Ils occupent une grande part de l'activité des services d’urgence et leur fréquence varie selon le contexte entre 11 et 30% [1-3,6]. Ces traumatismes sont à l'origine de simples lésions et de blessures graves [1-10]. Pratiquement non étudié dans notre contexte, ce premier travail avait pour but de rapporter les aspects sociodémographiques et étiopathogéniques des AD au Service des Urgences du Centre hospitalier national (CHN) El-Maarouf de Moroni en Union des Comores.

## Méthodes

**Type et cadre de l'étude:** nous avons mené une étude transversale descriptive à visée rétrospective sur une période de deux mois s'étendant du 1^er^ janvier au 28 février 2021 au Service des Urgences du Centre hospitalier national (CHN) El-Maarouf de Moroni, principal centre de référence de l'archipel de l'Union des Comores.

**Population d'étude:** la population d'étude était constituée de tous les enfants âgés de moins de 15 ans reçus et pris en charge au Service des Urgences du CHN El-Maarouf pour un accident domestique durant la période d'étude.

***Critères d'inclusion:*** nous avons inclus tous les enfants de moins de 15 ans reçus et pris en charge au service des urgences pour un AD ayant un dossier médical exploitable.

***Critères de non-inclusion:*** les enfants victimes d'autres types de traumatisme (accidents de la voie publique, accidents scolaires, sévices et maltraitance) et ceux âgés de plus de 15 ans ont été exclus de l'étude. Les dossiers médicaux incomplets ou mal renseignés, non exploitables, ont été exclus.

**Taille de l'échantillon:** l'échantillon était exhaustif. Tous les cas répondant aux critères d'inclusion durant la période de notre étude ont été retenus, sans calcul préalable de la taille.

**Variables étudiées:** nous avons étudié divers paramètres sociodémographiques tels que l'âge, le genre, la zone de résidence (urbaine, semi-urbaine et rurale) des patients et les caractéristiques de l'accident (la fréquence, le lieu de survenue, le type et le mécanisme).

**Sources de données et méthodes de collecte:** la collecte des données a été réalisée grâce à une fiche d'enquête anonyme et individuelle. Les données ont été recueillies à partir des dossiers médicaux des patients et des registres du Service des urgences du CHN El-Maarouf.

**Méthodes statistiques:** la saisie, le traitement et l'analyse des données ont été réalisés à l'aide des logiciels Microsoft Excel et Word. Les variables quantitatives ont été décrites par des moyennes, et les variables qualitatives par des effectifs et pourcentages. Aucune analyse analytique ou multivariée n'a été réalisée, l'étude étant purement descriptive.

**Considérations éthiques:** l'étude a respecté les principes d'éthique et de confidentialité. Les données collectées étaient anonymisées et utilisées uniquement à des fins scientifiques. Aucune information permettant d'identifier les patients n'a été recueillie.

**Gestion des biais:** afin de réduire les biais d'information, seuls les dossiers médicaux complets et exploitables ont été retenus pour l'analyse. Les variables d'intérêt ont été définies de manière précise avant le début de la collecte des données, et une fiche d'enquête standardisée a été utilisée afin d'assurer l'homogénéité et la fiabilité de la saisie des informations. Néanmoins, un biais de sous-déclaration ne peut être totalement exclu. Celui-ci est lié au caractère rétrospectif de l'étude, à la qualité variable du renseignement des dossiers médicaux, ainsi qu'au recours fréquent des populations aux structures de soins périphériques ou aux pratiques traditionnelles, susceptibles de limiter la captation exhaustive des accidents domestiques.

## Résultats

**Participants:** au cours de la période d'étude, 1995 passages ont été enregistrés au Service des urgences du CHN El-Maarouf. Parmi ceux-ci, 223 enfants ont été pris en charge pour un accident domestique, correspondant à une fréquence de 11,17%.

**Caractéristiques sociodémographiques des participants:** les garçons représentaient la majorité des victimes avec 65,02% des cas (n = 145) contre 34,98% de filles (n = 78), soit un sex-ratio de 1,08. L'âge moyen des enfants était de 7,07 ans. La tranche d'âge des 0 à 5 ans était la plus représentée avec 43,5% des cas, suivie des enfants âgés de 6 à 10 ans (29,6%) et de ceux de 11 à 15 ans (26,9%). Concernant l'origine géographique, la majorité des enfants provenait des zones suburbaines, notamment des régions de Bambao et d'Itsandra, représentant 42,15% des cas (n = 94). Les enfants issus des zones rurales et urbaines représentaient respectivement 29,6% et 28,25% des cas (Tableau 1).

**Tableau 1:** répartition des enfants selon l'origine géographique

Origines géographiques	Effectifs (n)	Pourcentage (%)
Régions suburbaines (Bambao et Itsandra)	94	42,15
Régions rurales	66	29,6
Moroni	63	28,25
**Total**	**223**	**100**

**Figure 1:**
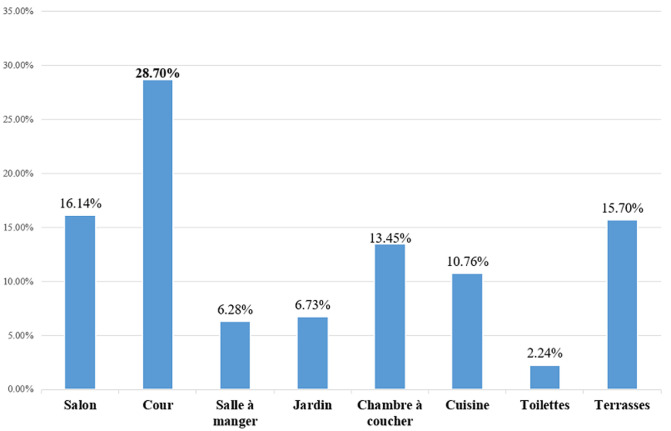
répartition des enfants selon le lieu de survenue de l'accident

**Lieu de survenue des accidents:** la cour de la maison constituait le lieu le plus accidentogène avec 28,70% des cas (n = 64), suivie du salon et de la terrasse. La Figure 1 présente la répartition des accidents domestiques selon leur lieu de survenue.

**Figure 2:**
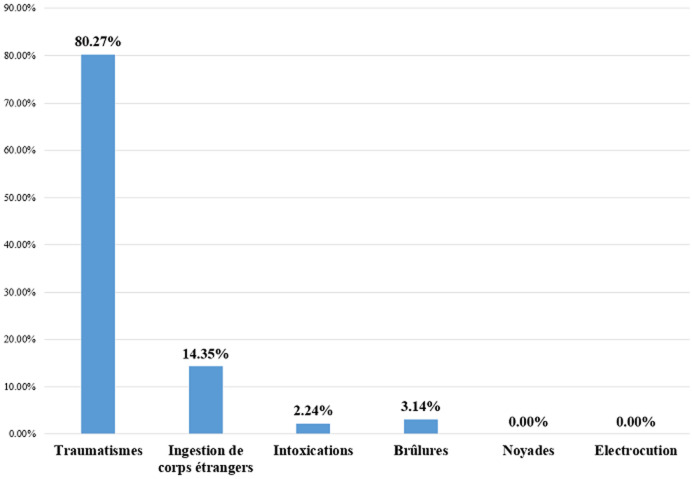
répartition des accidents domestiques selon le type d'accident

**Types d'accidents domestiques:** les traumatismes représentaient la principale catégorie d'accidents domestiques avec 80,27% des cas (n = 179). Ils étaient suivis par les ingestions de corps étrangers (14,35%) et les brûlures (3,14%). La répartition des accidents selon leur type est illustrée dans la Figure 2.

**Mécanismes des traumatismes:** parmi les traumatismes, la chute était le mécanisme prédominant avec 88,27% des cas (n = 158). Les objets piquants, tranchants et coupants représentaient 10,05% des cas (n = 18). Les autres mécanismes étaient rares. Le Tableau 2 détaille la répartition des traumatismes selon leur mécanisme.

**Tableau 2:** répartition des traumatismes selon le mécanisme

Mécanisme	Effectif (n)	Pourcentage (%)
Chute	158	88,27
Objets Piquants, Tranchants et Coupants (OPTC)	18	10,05
Autres	3	1,68
Total	179	100

**Caractéristiques spécifiques des ingestions de corps étrangers:** un total de 32 cas d'ingestion de corps étrangers a été enregistré, représentant 14,35% de l'ensemble des accidents domestiques. Dans la majorité des cas (n = 20), la nature du corps étranger était inconnue. Les objets métalliques et les pièces de monnaie représentaient chacun 18,75% des cas.

**Brûlures et intoxications:** sept cas de brûlures ont été rapportés. Elles étaient majoritairement dues à un liquide chaud (n = 4), suivies des brûlures par flamme (n = 3). Les intoxications représentaient 2,24% des cas (n = 5). Il s'agissait principalement d'ingestions de produits ménagers (n = 4) et d'un cas d'ingestion médicamenteuse.

## Discussion

Durant notre période d'étude, nous avons colligé 223 enfants victimes d'AD sur un total de 1995 consultations, soit un taux d'incidence quotidien de 3,7 AD. Les AD représentent donc une part non négligeable de l'activité de consultation au Service des urgences de CHN El-Maarouf. Notre chiffre est nettement supérieur à celui retrouvé au Mali avec 1,6% [11], au Gabon [12] avec 2,2% et au Sénégal avec 6,91% [1,3,6,13]. Au Sénégal, Mohamed avait trouvé un taux d'incidence supérieur à notre taux avec 28%. Car les consultations se faisaient directement dans un service d'urgences de chirurgie pédiatrique [3]. Notre fréquence est loin de refléter la réalité de l'épidémiologie des AD aux Comores. Les hôpitaux pôles et centres de santé implantés dans les différents districts sanitaires reçoivent une grande partie de ces AD.

Les enfants des régions de Bambao et Itsandra (autour de la capitale Moroni) étaient les plus représentés dans notre série avec 42,15% des cas. Les parts des victimes vivant en ville et en zone rurale étaient semblables avec respectivement 28,25% et 29,6%. Nous pouvons expliquer cela par la proximité et l'accessibilité des structures hospitalières en ville et dans les régions autour. Dans les autres régions, les enfants consultent dans les postes et centres de santé de district ou les parents ont recours aux gestes traditionnels (massage, application locale de produits à base de plantes) en cas de traumatisme. Il ressort de notre étude que les AD évoluent avec l'âge. La tranche d'âge des moins de 5 ans était la plus touchée avec 43,50% des cas. Ce résultat est inférieur à celui retrouvé au Sénégal avec 71,83%. Ce même phénomène est constaté dans plusieurs séries de la littérature [1,3,6,10-14]. Ces données peuvent s'expliquer par le fait que les enfants de bas âge sont très difficiles à surveiller à cause de leur curiosité, de leur autonomie et parce qu'ils passent beaucoup de temps à la maison. L'enfant va dans le monde du découvert et du danger [12].

Dans notre série, on notait une surreprésentation masculine avec 65,02%. Deux victimes sur trois étaient donc des garçons. Ce constat a été retrouvé par plusieurs auteurs [1-12]. Cette prédominance masculine s'expliquerait par le fait que les garçons se livrent le plus souvent à des jeux plus dangereux que les filles. Dans notre contexte, les filles ont plus d'attention parentale et passent la plupart du temps à apporter une assistance aux tâches ménagères. Ce qui explique en partie leur faible représentation. Les traumatismes arrivent largement en tête dans notre enquête, suivis des ingestions de CE puis des brûlures. Ce résultat est similaire à celui de Mohamed *et al*. au Sénégal, d’Ategbo au Gabon et de Touré *et al*. au Mali [1-3,6,10-12]. Dans notre travail, la chute est le principal mécanisme des traumatismes. Cela peut être expliqué par le fait que le jeu constitue la principale activité des enfants à domicile durant laquelle ils se livrent à des courses, des sauts et combats pouvant occasionner ces chutes. D'autres études placent également la chute comme principal mécanisme des AD par traumatismes [3,6,10-12]. Zwi *et al*. [13] dans son étude sur les AD dans les pays en développement ainsi que dans des séries européennes [13] placent la chute comme principal mécanisme des traumatismes.

L'ingestion de CE est en deuxième rang dans notre étude. Ce résultat est semblable à celui rapporté par Rafai *et al*. au Maroc [14]. Ce dernier a rapporté que 20 à 25% des inhalations de CE étaient dues à des cacahuètes et des corps végétaux puis suivies des objets de petite taille (pièce de monnaie, jetons, billes, …). Il montre aussi que la nature des corps étrangers est influencée par les conditions socioculturelles [14]. Les brûlures constituent dans notre série la 3^e^ cause des AD. Plusieurs auteurs [1,3,6-14] ont classé la brûlure parmi les trois premières causes des AD. Les brûlures par liquide bouillant et par flamme arrivent respectivement en première et deuxième position avec une nette prédominance des liquides chauds. Dans notre contexte, ce phénomène s'explique en partie par les conditions socioculturelles. Dans plusieurs ménages, les espaces dédiés à la cuisine ne sont pas sécurisés. Le bois et le pétrole lampant sont les principales sources utilisées en cuisine, ce qui augmente la fréquence des brûlures. Dans notre étude, la majorité des AD sont survenus dans la cour, lieux suivis du salon et de la terrasse. L'ordre de fréquence du lieu de survenue des AD est variable d'une étude à l'autre. Cependant, comparés à d'autres séries, nos résultats ont en commun le fait que la cour fait partie des trois principaux lieux de prédilection des AD chez l'enfant. Dans notre contexte, les alentours de la maison constituent l'aire de jeux préférée des enfants, ce qui explique en partie la grande survenue des traumatismes.

## Conclusion

Les AD chez l'enfant sont fréquents dans notre service et surviennent essentiellement dans la cour et au salon. Les victimes sont essentiellement des garçons âgés de moins de 5 ans. Il s'agissait le plus souvent de traumatismes ou d'ingestion de CE. Dans notre contexte, le manque d'aires de jeux aménagées, des habitations ne répondant pas aux normes de sécurité et la surveillance insuffisante des enfants sont des facteurs particuliers influençant la fréquence des AD. La courte durée de notre étude ne nous a pas permis d'explorer notre travail et les dossiers médicaux étaient mal renseignés, ce qui fait de notre étude un prélude laissant le champ à plusieurs travaux scientifiques prospectifs et analytiques qui tiendront compte de plusieurs paramètres sociodémographiques et cliniques.

### Etat des connaissances sur le sujet

- Les AD sont majoritaires des accidents de la vie courante, le plus souvent mortels en Europe avec 120 000 décès chaque année;
- Les enfants de moins de 5 ans sont souvent victimes d'AD et davantage ceux âgés de 1 à 5 ans;
- La chute représente le principal mécanisme mis en cause dans ces accidents qui sont le plus souvent à l'origine de contusions et de plaies.

### Contribution de notre étude à la connaissance

- Notre étude confirme la grande fréquence des AD chez les petits enfants et la prédominance masculine;
- Dans notre contexte, les traumatismes par chute à domicile étaient le type d'accident le plus fréquent, suivis des ingestions de corps étrangers;
- Notre étude se veut le prélude à d'autres études de grande envergure permettant de faire d'utiles recommandations pour la prévention des accidents domestiques à l'échelle nationale.

## Remerciements

Nous remercions les enfants et leurs familles pour leur participation volontaire ainsi que le personnel du Service des urgences du CHN El-Maarouf pour leur contribution à cette étude.
